# Solanaceous Vegetables and Colorectal Cancer Risk: A Hospital-Based Matched Case-Control Study in Northeast China

**DOI:** 10.3389/fnut.2021.688897

**Published:** 2021-07-12

**Authors:** Yang Liu, Simin Li, Liqing Jiang, Yuchong Zhang, Zhi Li, Jing Shi

**Affiliations:** ^1^Department of Medical Oncology, The First Hospital, China Medical University, Shenyang, China; ^2^Department of Clinical Epidemiology and Center of Evidence-Based Medicine, The First Affiliated Hospital, China Medical University, Shenyang, China

**Keywords:** solanaceous vegetables, colorectal cancer, case-control study, Northeast China, hospital-based

## Abstract

**Background:** Dietary factors are regarded as an essential influence in changing colorectal cancer (CRC) risk. However, there is no clear conclusion of the relationship between solanaceous vegetables and colorectal cancer at present. The study aimed to evaluate the intake of solanaceous vegetables in relation to colorectal cancer risk among the Northeast Chinese population.

**Methods:** We carried out a hospital-based case-control study in three hospitals in Northeast China from 2009 to 2011. The study finally included 833 patients with CRC and 833 controls matched separately according to age, gender, and city of residence. We applied a structural questionnaire to collect demographic characteristics and dietary information by face-to-face interview and adopted conditional logistic regression to calculate odds ratios (ORs) and 95% confidence intervals (CIs). Stratified analyses were conducted by sex and subsites.

**Results:** There was no obvious correlation between total intake of solanaceous plants and CRC risk. The adjusted OR for the highest quartile and the lowest quartile was 1 (95% CI: 0.68–1.5). Certain types of solanaceous vegetables were negatively associated with the risk of CRC, such as eggplant (OR = 0.42; 95% CI:0.29–0.62) and sweet pepper (OR = 0.48; 95%CI: 0.33–0.7). Potato was found to have a positive correlation with CRC (OR = 1.76; 95% CI: 1.26–2.47). In the stratified analyses by gender, total solanaceous vegetables intake was inversely associated with CRC risk only in men. In the stratified analyses of cancer subsites, no significant association between total solanaceous vegetables intake and CRC risk was found.

**Conclusion:** No findings showed that the intake of total solanaceous vegetables was related to the reduction of CRC risk. However, specific types of solanaceous vegetables indicated an inverse association with CRC risk.

## Background

Colorectal cancer (CRC) is the third most common malignant tumor in the world and is also the second primary cause of death from cancer worldwide. In 2020, there were nearly 1.9 million new CRC cases (10%), and almost 0.9 million CRC patients died (9.4%) (1). The incidence of and mortality from CRC in China are lower compared with the world average (incidence rate: 1,781 per 100,000 people; mortality rate: 8.12 per 100,000 people), but the number of new CRC cases and CRC-related deaths in China is the highest in the world (2).

Environmental and genetic factors play a primary role in the pathogenesis of CRC. Evidence shows that lifestyles, which include diet, drinking, smoking, physical activity, body mass index (BMI), and sleep are also related to the CRC risk (3–6), while the dietary factors are regarded as a vital source of changing CRC risk. It is commonly found in studies that the intake of red meat, processed meat, animal fat, and sugar may be related to increased CRC risk (7–12), while the high consumption of fruits, vegetables, fish, and some nutrients may prevent the development of CRC (13–17).

Solanaceous vegetables (mainly including eggplants, peppers, tomatoes, and potatoes) are consumed worldwide because of their high yield and easy storage. However, there are few studies of the relationship between consumption of solanaceous vegetables and colorectal cancer risk at present, and the results have not reached a clear conclusion. A study on Norwegian women indicated a positive association between high potato intake and CRC risk (HR: 1.32, 95% CI: 1.1, 1.6 for ≥3 potatoes per day vs. 0–7 potatoes per week) (18). Another case-control study on colorectal cancer in Italy confirmed that tomato intake was significantly protective against CRC after controlling for potential confounding factors (ORs for the highest intake quartile were.79 (95% CI.6–0.9) for colon cancer, and 0.71 (95% CI 0.5–0.9) for rectal cancer) (19).

Certain unique and common chemical components in *Solanaceae* are proved to have an anti-cancer effect. On one hand, all solanaceous vegetables have anthocyanin with a strong anti-cancer effect because of the anti-oxidation mechanism (20). A meta-analysis showed that the consumption of anthocyanin is inversely proportional to the CRC risk, which may play a positive role in the prevention of CRC (21). Additionally, glycoalkaloids are a nitrogenous secondary metabolic product discovered in many solanaceous plants. The exposure of cancer cells to carbohydrate alkaloid derivatives and aglycons can inhibit cell growth in the culture (*in vitro*) and the growth of *in vivo* tumors (22). Experiments show that carbohydrate alkaloids and their metabolites can inhibit human colon cancer cells and hepatoma cells (23).

The increase in CRC cases in China is closely related to gradually westernized lifestyle (24, 25). Nevertheless, the vegetable intake of China (293.1 g/day) is still higher than that of the world average (208.8 g/day) and that of most Western countries (26). Solanaceous vegetables are consumed in great abundance among the Northeast Chinese population. However, the association between dietary intake of solanaceous vegetables and CRC risk in Chinese population remained inconclusive. Therefore, we carried out a hospital-based matching case-control study in three general hospitals in Northeast China in order to clarify the relationship.

## Materials and Methods

### Ethical Statement

The study was approved by the Human Research Ethics Committees of the three hospitals. The moral code is [2009] 12. All of the participants in the study have provided written informed consent before the investigation.

### Research Objects

Patients of 18 years old or above who received treatment in China Medical University, Shengjing Hospital of China Medical University, and the First Affiliated Hospital of Dalian Medical University from June 17, 2009, to November 5, 2011, and who were diagnosed as having primary CRC are qualified to participate in the research. Patients in the control group were randomly selected from the physical examination center of the same hospital and matched with the case group according to age (within 5 years old), gender and residential district (1:1). Exclusion criteria: participants with antecedent cancers, glucocorticoid-treated immune system disorders, cardiovascular disease, mental disorder, chronic digestive system diseases, benign colorectal damages, and other conditions that might lead to long-term diet alteration were excluded from this study (27). Data cleaning was performed by deleting subjects with any missing value and cases (controls) with missing matched controls (cases).

### Data Collection

Specially trained personnel were arranged to carry out a structural questionnaire to the studied participants through a face-to-face interview. The trained personnel assessed the regular diet of the participants as adults within a year before the interview, and the participants were asked whether they had changed their dietary habits. The questionnaire required the participants to answer the questions according to their dietary habits before any change, if there is. The interview usually lasted 40–50 min. The case group had to complete the questionnaire within 120 days from the day of diagnosis. The control group accepted the interview within 3 months after the corresponding participants. The following data were collected: (1) general demographic characteristics, for example, education level, family history, height, and weight; (2) lifestyle factors and dietary habits, for example, smoking and alcohol status, breakfast habit, and intake of fried food; and (3) frequency and quantity of 99 common foods. After each interview, the trained investigators inspected the integrity and authenticity of the data. The annual intake of each food was calculated as food consumption (50 g) multiplied by intake frequency, expressed by kg/year. The total intake of solanaceous vegetables was computed as the totality of the annual intake of potato, tomato, eggplant, and sweet pepper. Intake of the total energy was calculated according to the Ingredient List of Chinese Foods. The body mass index (BMI) of each participant was calculated by the weight (in kg) 5 years before the interview divided by the square of height (m^2^). The weight and height were measured by the hospitals. The patients were then divided into three BMI groups (<25, 25–30, and >30). Dietary intake of 25 nutrients was calculated according to the 2004 Chinese Food Composition Table (28).

### Statistical Analysis

SPSS19.0 was used for all statistical analyses. The differences in general conditions and dietary factors between the two groups were calculated by χ2 test (categorical variables) and t-test (continuous variables). Since the data distribution was skewed, the Mann–Whitney *U* test estimated differences in selected dietary intake. According to the distribution of the control group, solanaceous vegetable intake and nutrient intake data were classified into quartiles, and the lowest quartile was used as a reference. A conditional logistic regression model was used to calculate the crude and adjusted ORs and 95% CIs for the association between solanaceous vegetables, nutrient intake, and CRC risk. Tests for trends were calculated with the median intake of each quartile of solanaceous vegetable intake and nutrients intake as a continuous variable. In the multivariate logistic regression analyses, we performed multicollinearity diagnostics by calculating the variance inflation factor (VIF) to avoid multicollinearity problems among variables, and VIF > 10 is considered to have multicollinearity. Variables with VIF > 10 were deleted. Based on literature, BMI, an education level (below high school/above high school), history of CRC in first-degree relatives (none/yes), smoking status (total > 20 packs are considered smoking), drinking status, total energy intake, red and white meat intake, egg intake, milk product intake, total vegetable intake, total fruit intake, solanaceous vegetable intake, non-solanaceous vegetable intake, and dietary habits were selected as potential confounding factors in the regression models for the association between solanaceous vegetables and CRC risk. As for the association of nutrients intake and CRC risk, we included BMI, colon cancer in first-degree relative, smoking, drinking, total energy intake, and red meat intake in the final multivariate regression models. No multicollinearity was detected among the variables mentioned above. Finally, we conducted subgroup analyses according to CRC subsites and gender. *P* < 0.05 is considered statistically significant.

## Results

### General Characteristics

One thousand eighty-two cases and 1,025 controls were qualified for this study, and 943 cases and 945 controls agreed to participate. After data cleaning, a total of 833 cases and 833 controls matched and were finally involved in the research. Results of the general characteristics of the participants are shown in Table 1. Compared with the control group, the first-degree relatives of case group had more patients with colorectal cancer history (*P* < 0.001), and smokers (*P* < 0.001) and drinkers (*P* = 0.005) were also more in the case group. However, no significant differences in the BMI and educational background were found between the case group and the control group.

**Table 1:** General characteristics of all subjects.

**General characteristics**	**Case (*n* = 833) n%**	**Control (*n* = 833) n%**	*P*-value^*^
**Age**	60 (53,67)	60 (53,66)	
**Gender**			
Male	483 (58.0%)	483 (58.0%)	
Female	350 (42%)	350 (42%)	
**BMI**			0.305
<25	539 (64.7%)	568 (68.2%)	
25–30	259 (31.1%)	231 (27.7%)	
>30	35 (4.2%)	34 (4.1%)	
**Family history of colorectal cancer (first degree)**			**<0.001**
No	784 (94.1%)	825 (99.0%)	
Yes	49 (5.9%)	8 (1.0%)	
**Education level**			0.654
< senior	489 (58.7%)	498 (59.8%)	
>senior	344 (41.3%)	335 (40.2%)	
**Alcohol drinking**			**0.005**
never	518 (62.2%)	572 (68.7%)	
ever	315 (37.8%)	261 (31.3%)	
**Smoking status**			**<0.001**
no	489 (58.7%)	575 (69.0%)	
yes	344 (41.3%)	258 (31.0%)	

* *P-value for chi-square test*.

*Bold values indicates statistical significance, which is defined as a two-side P < 0.05*.

### Dietary Habits and Selected Dietary Intake

The dietary habits of the patients and the medians (the 25th percentiles and the 75th percentiles) of selected dietary intake are shown in Table 2. Compared with the control group, the case group had fewer people taking breakfast regularly (*P* < 0.001) but more people eating barbecue, and fried, hot, and spicy foods (*P* < 0.001). In addition, significant differences were also observed in the selected dietary intake between the case group and the control group: total energy and red meat of the case group were higher than those of the control group; while the intake of white meat, eggs, dairy products, total intake of fruits, non-solanaceous vegetables as well as solanaceous vegetables of the case group were all lower than those of the control group. In the four separately evaluated solanaceous vegetables, consumptions of tomato, eggplant, and sweet pepper in the case group were obviously reduced, while the intake median of potato in the case group was higher compared with that in the control group.

**Table 2:** Dietary habits and selected dietary intake of all subjects.

**Dietary habits**	**Case (*n* = 833)**	**Control (*n* = 833)**	*P*-value^*^
**Eat breakfast**			**<0.001**
never	70 (8.4%)	27 (3.2%)	
Sometimes	158 (19.0%)	58 (7.0%)	
often	605 (72.6%)	748 (89.8%)	
**Fried food intake**			**<0.001**
often	41 (4.9%)	8 (1.0%)	
sometimes	72 (8.6%)	34 (4.1%)	
Occasionally	293 (35.2%)	262 (31.5%)	
never	427 (51.3%)	529 (63.5%)	
**Cured food intake**			**<0.001**
Often	41 (4.9%)	15 (1.8%)	
Sometimes	57 (6.8%)	30 (3.6%)	
Occasionally	277 (33.3%)	202 (24.2%)	
never	458 (55.0%)	586 (70.3%)	
**Hot and spicy food intake**			**<0.001**
Hot and spicy	178 (21.4%)	53 (6.4%)	
Either hor or spicy	34 (4.1%)	37 (4.4%)	
Neither hot or spicy	621 (74.5%)	743 (89.2%)	
**Yearly dietary intake**			***P***-value^**^
Red meats (kg/year)	20.70 (8.20,38.63)^a^	13.40 (7.70,21.73)	**<0.001**
White meats (kg/year)	7.20 (3.60,14.55)	9.60 (5.25,15.38)	**<0.001**
Eggs (kg/year)	6.50 (2.60,18.25)	13.00 (6.50,18.25)	**<0.001**
Milk product (kg/year)	1.50 (0.00,32.50)	13.00 (0.00,54.75)	**<0.001**
Potato (kg/year)	9.75 (4.20,13.00)	7.80 (3.90,13.00)	0.247
Tomato (kg/year)	5.20 (3.00,13.00)	6.50 (3.75,16.25)	**<0.001**
Eggplant (kg/year)	5.20 (3.00,13.00)	7.80 (5.20,16.25)	**<0.001**
Sweet pepper (kg/year)	2.25 (0.30,4.88)	2.60 (0.75,5.20)	**0.001**
Total fruits (kg/year)	59.50 (23.85,121.53)	93.00 (47.65,155.50)	**<0.001**
Total energy (MJ/year)	2,313.94 (1,669.83, 3,154.11)	2,163.20 (1,641.12, 2,838.49)	**0.004**
Total solanaceous vegetables (kg/year)	26.25 (17.18,42.90)	31.20 (19.10,48.75)	**<0.001**
Total non-solanaceous vegetables (kg/year)	92.89 (68.35,126.28)	112.95 (80.25,153.99)	**<0.001**

* *P-value for chi-square test*.

** *P-value for Mann–Whitney U test*.

a *Median (25th and 75th percentiles)*.

*Bold values indicates statistical significance, which is defined as a two-side P < 0.05*.

### Consumption of Solanaceous Vegetables and CRC Risk

We obtained the CRC risk ORs and 95% CIs related to the intake of four specific solanaceous vegetables and the total solanaceous vegetables (Table 3) based on the univariate and multivariate logistic regression analyses. In the univariate analyses, the results indicated that the intakes of total solanaceous vegetables, tomato, eggplant, and sweet pepper were inversely related to CRC risk. After adjustment, however, only the intakes of eggplant and sweet pepper showed a negative association with CRC risk, and the ORs (and 95% CIs) for Q4 vs. Q1 were 0.42 (0.29–0.62) and 0.48 (0.33–0.7), respectively. In addition, there was no significant correlation between the intake of potato and the CRC risk (*P* = 0.293) in the univariate analysis, but after adjustment, the OR for the maximum quartile, compared with the minimum quartile of potato intake, was 1.76 (95% CI = 1.26–2.47, *P* = 0.007), suggesting that higher intake of potato was associated with increased risk of CRC.

**Table 3:** Odds ratios (ORs) and 95% confidence intervals (CIs) for the association between solanaceous vegetables intake and colorectal cancer risk.

**Yearly solanaceous vegetables intake quartiles (kg/year)**	**Case**	**Control**	**Crude OR (95%CI)**	**Adjusted OR^**a**^ (95%CI)**
**Potato**				
Q1	164	196	1.00	1.00
Q2	217	211	1.22 (0.93,1.61)	1.23 (0.85,1.76)
Q3	53	52	1.22 (0.79,1.89)	1.43 (0.81,2.51)
Q4	399	374	1.28 (0.99,1.65)	1.76 (1.26,2.47)
P for Trend^b^			0.293	**0.007**
**Eggplant**				
Q1	302	177	1.00	1.00
Q2	192	189	0.62 (0.48,0.82)	0.65 (0.46,0.92)
Q3	193	208	0.49 (0.37,0.66)	0.71 (0.49,1.01)
Q4	146	259	0.29 (0.21,0.39)	0.42 (0.29,0.62)
P for Trend			**< 0.001**	**<0.001**
**Sweet pepper**				
Q1	245	172	1.00	1.00
Q2	205	214	0.66 (0.50,0.88)	0.54 (0.37,0.78)
Q3	176	208	0.59 (0.45,0.79)	0.52 (0.35,0.75)
Q4	207	239	0.58 (0.44,0.78)	0.48 (0.33,0.70)
P for Trend			**<0.001**	**<0.001**
**Tomato**				
Q1	260	188	1.00	1.00
Q2	197	194	0.73 (0.55,0.96)	0.73 (0.51,1.03)
Q3	219	215	0.72 (0.55,0.94)	0.76 (0.53,1.08)
Q4	157	236	0.43 (0.32,0.58)	0.59 (0.40,0.88)
P for Trend			**<0.001**	0.066
**Total solanaceous vegetables**				
Q1	253	208	1.00	1.00
Q2	229	208	0.92 (0.71,1.20)	0.96 (0.68,1.35)
Q3	185	208	0.71 (0.54,0.93)	0.97 (0.68,1.40)
Q4	106	209	0.60 (0.45,0.81)	1.00 (0.68,1.50)
P for Trend			**0.003**	0.993
**Total non-solanaceous vegetables**				
Q1	317	207	1.00	1.00
Q2	234	210	0.69 (0.53,0.91)	0.80 (0.57,1.12)
Q3	163	208	0.48 (0.36,0.64)	0.63 (0.43,0.91)
Q4	119	208	0.35 (0.26,0.48)	0.51 (0.34,0.77)
P for trend			**<0.001**	**0.009**

a *Adjusted by BMI, colon cancer in first-degree relative, smoking status, alcohol drinking, eating breakfast, and fried food, grilled food, and hot and spicy food intake as category variables; and total energy, total fruits, milk product and red meat intake as continuous variables*.

b *Test for trends calculated with the median intake of each category of solanaceous vegetable intake as a continuous variable*.

*Bold values indicates statistical significance, which is defined as a two-side P < 0.05*.

### Subgroup Analysis

We further discussed the association of the intake of solanaceous vegetables and CRC risk by different subsites (Table 4). In the proximal colon cancer subgroup, eggplant and tomato intakes were found to be inversely correlated with the risk of proximal colon cancer in the univariate analyses, and the ORs for Q4 vs. Q1 were 0.2 (95% CI = 0.1–0.65, *P* < 0.001) and 0.42 (95% CI = 0.23–.78, *P* = 0.006), respectively. However, the correlation between tomato consumption and the risk of colon cancer was no longer significant after adjustment (*P* = 0.098). In the distal colon cancer subgroup, despite a negative correlation between tomato intake and risk of colon cancer in the univariate analyses, no obvious association was found after adjustments in the multivariate analyses. The OR for Q4 vs. Q1 was 0.39 (95% CI = 0.2–0.77, *P* = 0.032). In the rectal cancer group, the eggplant and sweet pepper intakes were observed to be related to a decrease in the risk of cancer, and the adjusted ORs for Q4 vs. Q1 were 0.32 (95% CI = 0.18–0.56, *P* = 0.001) and 0.47 (95% CI = 0.26–0.85, *P* = 0.019), respectively, while the intake of tomato only showed a significantly inverse correlation with rectal cancer risk before adjustment with an OR for Q4 vs. Q1 of 0.45 (95% CI = 0.3–0.68, *P* = 0.002). Stratified analyses by gender showed that in the male subgroup, increased eggplant and sweet pepper consumptions were significantly correlated with reduction of the CRC risk both in the univariate and multivariate regression models, while the intakes of total solanaceous vegetables and tomato were inversely related to the CRC risk only in the univariate analyses. In the female subgroup, eggplant and sweet pepper intakes were inversely associated with the CRC risk in the multivariate analyses (Table 5).

**Table 4:** Odds ratios (ORs) and 95% confidence intervals (CIs) for the association of yearly solanaceous vegetables intake and colorectal cancer risk according to subsite.

**Yearly solanaceous vegetables intake quartiles**	**Proximal colon cancer (*****n*** **=** **396)**			**Distal colon cancer (*****n*** **=** **418)**			**Rectal cancer (*****n*** **=** **852)**		
	**Cases/ controls (*n*)**	**OR (95%CI)**	**Adjusted OR^a^ (95%CI)**	**Cases/ controls (*n*)**	**OR (95%CI)**	**Adjusted OR (95%CI)**	**Cases/ controls (*n*)**	**OR (95%CI)**	**Adjusted OR (95%CI)**
**Potato**									
Q1	37/48	1.00	1.00	46/46	1.00	1.00	81/102	1.00	1.00
Q2	52/57	1.16 (0.67,2.01)	1.58 (0.75,3.35)	60/55	1.06 (0.62,1.83)	0.87 (0.38,1.96)	105/99	1.34 (0.90,2.00)	1.28 (0.74,2.23)
Q3	12/12	1.23 (0.52,2.88)	2.80 (0.81,9.75)	9/14	0.63 (0.24,1.66)	0.66 (0.16,2.71)	32/26	1.58 (0.86,2.92)	1.81 (0.80,4.08)
Q4	97/81	1.51 (0.91,2.50)	3.22 (1.54,6.71)	94/94	0.98 (0.58,1.68)	1.81 (0.82,4.00)	208/199	1.31 (0.93,1.87)	1.36 (0.85,2.19)
P for Trend^b^		0.412	**0.013**		0.733	0.119		0.330	0.454
**Eggplant**									
Q1	74/47	1.00	1.00	65/65	1.00	1.00	163/85	1.00	1.00
Q2	48/34	0.90 (0.48,1.67)	0.83 (0.37,1.85)	45/59	0.54 (0.32,0.93)	0.60 (0.27,1.35)	99/96	0.59 (0.41,0.85)	0.55 (0.34,0.90)
Q3	46/44	0.59 (0.32,1.09)	0.66 (0.31,1.41)	53/50	0.73 (0.41,1.28)	1.10 (0.49,2.44)	94/114	0.39 (0.26,0.59)	0.57 (0.34,0.98)
Q4	30/73	0.20 (0.10,0.39)	0.25 (0.10,0.65)	46/55	0.57 (0.32,1.03)	1.06 (0.47,2.40)	70/131	0.24 (0.16,0.37)	0.32 (0.18,0.56)
P for Trend		**<0.001**	**0.03**		0.111	0.441		**<0.001**	**0.001**
**Sweet Pepper**									
Q1	55/39	1.00	1.00	66/43	1.00	1.00	124/90	1.00	1.00
Q2	52/46	0.86 (0.48,1.55)	0.65 (0.29,1.46)	45/51	0.54 (0.29,1.99)	0.39 (0.16,0.97)	108/117	0.66 (0.44,0.97)	0.47 (0.27,0.82)
Q3	41/57	0.53 (0.30,0.93)	0.41 (0.19,0.93)	43/46	0.57 (0.31,1.04)	0.52 (0.21,1.25)	92/105	0.64 (0.43,0.94)	0.50 (0.29,0.87)
Q4	50/56	0.64 (0.36,1.14)	0.48 (0.21,1.09)	55/69	0.48 (0.27,0.84)	0.39 (0.17,0.89)	102/114	0.62 (0.41,0.95)	0.47 (0.26,0.85)
P for Trend		0.125	0.146		0.062	0.114		0.059	**0.019**
**Tomato**									
Q1	57/51	1.00	1.00	69/45	1.00	1.00	134/92	1.00	1.00
Q2	46/38	1.10 (0.62,1.95)	0.89 (0.40,2.01)	43/53	0.56 (0.33,0.95)	0.69 (0.34,1.38)	108/103	0.70 (0.47,1.03)	0.73 (0.43,1.24)
Q3	56/41	1.22 (0.69,2.16)	1.68 (0.74,3.81)	62/62	0.62 (0.37,1.04)	0.71 (0.35,1.42)	101/112	0.60 (0.40,0.90)	0.60 (0.35,1.03)
Q4	39/68	0.42 (0.23,0.78)	0.57 (0.22,1.48)	35/49	0.39 (0.20,0.77)	0.85 (0.33,2.16)	83/119	0.45 (0.30,0.68)	0.57 (0.33,1.01)
P for Trend		**0.006**	0.098		**0.032**	0.680		**0.002**	0.183
**Total solanaceous vegetables**									
Q1	62/52	1.00	1.00	68/54	1.00	1.00	123/102	1.00	1.00
Q2	51/48	0.93 (0.55,1.59)	1.83 (0.85,3.93)	57/50	0.93 (0.55,1.57)	0.81 (0.39,1.71)	121/110	0.92 (0.64,1.33)	0.82 (0.49,1.39)
Q3	46/40	0.92 (0.52,1.61)	1.68 (0.76,3.72)	46/54	0.65 (0.38,1.11)	1.20 (0.56,2.59)	93/114	0.65 (0.44,0.96)	0.69 (0.39,1.21)
Q4	39/58	0.49 (0.26,0.90)	1.52 (0.59,3.89)	38/51	0.52 (0.29,0.96)	1.22 (0.53,2.82)	89/100	0.70 (0.46,1.06)	0.91 (0.51,1.63)
P for Trend		0.111	0.414		0.143	0.785		0.117	0.590

a *Adjusted by BMI, colon cancer in first-degree relative, smoking status, alcohol drinking, eating breakfast, and fried food, grilled food, and hot and spicy food intake as category variables; and total energy, total fruits, milk product, and red meat intake as continuous variables*.

b *Test for trends calculated with the median intake of each category of solanaceous vegetable intake as a continuous variable*.

*Bold values indicates statistical significance, which is defined as a two-side P < 0.05*.

**Table 5:** Odds ratios (ORs) and 95% confidence intervals (CIs) for the association of yearly solanaceous vegetables intake and colorectal cancer risk according to gender.

**Yearly solanaceous vegetables intake quartiles**	**Men (*****n*** **=** **966)**			**Women (*****n*** **=** **700)**		
	**Cases/ controls (n)**	**OR (95%CI)**	**Adjusted OR^a^ (95%CI)**	**Cases/ controls (n)**	**OR (95%CI)**	**Adjusted OR (95%CI)**
**Potato**						
Q1	101/114	1.00	1.00	63/82	1.00	1.00
Q2	126/119	1.19 (0.83,1.70)	1.16 (0.73,1.84)	91/92	1.29 (0.83,1.99)	1.38 (0.73,2.58)
Q3	32/28	1.29 (0.73,2.28)	1.39 (0.67,2.87)	21/24	1.10 (0.55,2.19)	1.84 (0.70,4.85)
Q4	224/222	1.14 (0.82,1.59)	1.52 (0.99,2.34)	175/152	1.50 (1.01,2.22)	2.41 (1.35,4.33)
P for Trend^b^		0.746	0.255		0.232	**0.018**
**Eggplant**						
Q1	181/101	1.00	1.00	121/76	1.00	1.00
Q2	103/101	0.60 (0.42,0.87)	0.65 (0.41,1.02)	89/88	0.65 (0.44,0.97)	0.65 (0.37,1.13)
Q3	114/119	0.50 (0.35,0.73)	0.71 (0.45,1.12)	79/89	0.49 (0.31,0.76)	0.65 (0.35,1.20)
Q4	85/102	0.25 (0.17,0.38)	0.39 (0.24,0.65)	61/97	0.34 (0.22,0.55)	0.47 (0.24,0.89)
P for Trend		**<0.001**	**0.003**		**<0.001**	**0.129**
**Sweet Pepper**						
Q1	142/94	1.00	1.00	103/78	1.00	1.00
Q2	109/117	0.60 (0.41,0.89)	0.49 (0.30,0.82)	96/97	0.74 (0.48,1.14)	0.57 (0.31,1.05)
Q3	109/121	0.59 (0.40,0.85)	0.59 (0.36,0.96)	67/87	0.59 (0.38,0.91)	0.40 (0.22,0.74)
Q4	123/151	0.49 (0.33,0.73)	0.51 (0.31,0.83)	84/88	0.73 (0.47,1.12)	0.39 (0.20,0.76)
P for Trend		**0.003**	**0.018**		0.115	**0.011**
**Tomato**						
Q1	143/101	1.00	1.00	117/87	1.00	1.00
Q2	113/110	0.74 (0.51,1.06)	0.91 (0.57,1.43)	84/84	0.72 (0.47,1.09)	0.54 (0.29,1.00)
Q3	134/129	0.74 (0.52,1.06)	0.89 (0.57,1.39)	85/86	0.68 (0.44,1.05)	0.56 (0.30,1.04)
Q4	93/143	0.42 (0.28,0.62)	0.70 (0.42,1.17)	64/93	0.45 (0.28,0.72)	0.46 (0.24,0.90)
P for Trend		**<0.001**	0.592		**0.012**	0.103
**Total solanaceous vegetables**						
Q1	151/115	1.00	1.00	102/93	1.00	1.00
Q2	123/120	0.79 (0.56,1.12)	0.78 (0.50,1.22)	106/88	1.13 (0.76,1.68)	1.45 (0.81,2.59)
Q3	109/116	0.69 (0.48,1.01)	0.99 (0.60,1.61)	76/92	0.72 (0.48,1.10)	1.14 (0.63,2.05)
Q4	100/132	0.52 (0.35,0.77)	0.92 (0.55,1.54)	66/77	0.72 (0.45,1.13)	1.24 (0.63,2.43)
P for Trend		**0.013**	0.698		0.146	0.658

a *Adjusted by BMI, colon cancer in first-degree relative, smoking status, alcohol drinking, eating breakfast, and fried food, grilled food, and hot and spicy food intake as category variables; and total energy, total fruits, milk product, and red meat intake as continuous variables*.

b *Test for trends calculated with the median intake of each category of solanaceous vegetable intake as a continuous variable*.

*Bold values indicates statistical significance, which is defined as a two-side P < 0.05*.

### Nutrients Intake and CRC Risk

We compared the daily intake of nutrients from solanaceous vegetables in the case group with that of the control group (Table 6) based on a *t*-test. The results showed that the intake of the following 20 nutrients of the CRC group was significantly lower than that of the control group, namely protein, fat, dietary fiber, vitamins A, E, C, and B2, folate, niacin, carotene, potassium, magnesium, sodium, calcium, iron, zinc, copper, iodine, manganese, and phosphorus.

**Table 6:** Comparison of nutrient intake between case group and control group.

**Nutrients**	**Case group x ± s**	**Control group x ± s**	***T*-value**	*P*-value^*^
Energy/kcal	1,2316.67 ± 9,423.33	12,739.07 ± 9,125.73	0.929	0.353
Protein/g	488.1 ± 362.68	523.62 ± 366.56	1.988	**0.047**
Fat/g	66.55 ± 49.81	77.03 ± 53.79	4.127	**<0.001**
Dietary fiber/g	523.67 ± 404.37	622.23 ± 450.09	4.702	**<0.001**
Carbohydrate/g	2,951.95 ± 2219.76	3,097.36 ± 2,184.58	1.348	0.178
Vitamin A/mg	6.95 ± 7.42	8.88 ± 9.46	4.654	**<0.001**
Vitamin E/mg	53.66 ± 56.91	67.19 ± 67.21	4.437	**<0.001**
Vitamin C/mg	7,169.8 ± 7,721.6	7,998.65 ± 6,979.39	2.298	**0.022**
Vitamin B6/mg	44.13 ± 33.89	45.82 ± 32.77	1.038	0.299
Folate/mg	2.95 ± 2.14	3.31 ± 2.25	3.309	**0.001**
Niacin/mg	104.66 ± 93.24	132.94 ± 103.49	5.859	**<0.001**
Carotene/mg	41.23 ± 44.09	52.74 ± 56.24	4.648	**<0.001**
Vitamin B1/mg	16.7 ± 12.55	17.56 ± 12.4	1.405	0.160
Vitamin B2/mg	5.69 ± 4.16	6.46 ± 4.26	3.703	**<0.001**
Potassium/mg	75,229.53 ± 55,385.67	82,862.7 ± 58,035.13	2.746	**0.006**
Magnesium/mg	5,185.15 ± 3,820.41	5,695.89 ± 3,971.29	2.675	**0.008**
Sodium/mg	2,126.71 ± 1,682.28	2,500.35 ± 1,951.7	4.185	**<0.001**
Calcium/mg	2,268.52 ± 1,719.59	2,577.58 ± 1,702.18	3.687	**<0.001**
Iron/mg	117.9 ± 89.83	132.18 ± 87.81	3.282	**0.001**
Zinc/mg	62.78 ± 46.15	68.34 ± 46.79	2.442	**0.015**
Copper/mg	20.65 ± 15.26	22.66 ± 15.34	2.686	**0.007**
Iodium/ug	476.13 ± 389.99	568.69 ± 468.82	4.381	**<0.001**
Manganese/mg	30.08 ± 22.16	34.26 ± 22.75	3.802	**<0.001**
Selenium/ug	80.47 ± 60.85	83.44 ± 56.87	1.031	0.303
Phosphorus/mg	9,562.3 ± 7,127.92	10,450.97 ± 7,438.32	2.49	**0.013**

* *P-value for t test*.

*Bold values indicates statistical significance, which is defined as a two-side P < 0.05*.

Univariate and multivariate logistic regression analyses were performed to investigate the relationship between the intake of nutrients and CRC risk. The results of ORs and 95% CIs are displayed in Table 7. In the univariate logistic regression analyses, significant inverse associations were found between the risk of CRC and intake of dietary fiber, vitamin A, vitamin E, vitamin C, folate, carotene, vitamin B2, calcium, and iodine. Furthermore, compared with the minimum nutrient intake group, the CRC risk of the three higher intake groups were significantly reduced, and the risk of CRC gradually decreased along with the increase in nutrient intake (*P* trend all <0.05). After controlling for the effects of suspected confounding factors, such as BMI, history of colon cancer in first-degree relative, smoking and drinking status, and intake of total energy and red meat, the results of the multivariate logistic regression analyses were consistent with those of the univariate logistic regression analyses. However, the adjusted OR of folate intake was no longer statistically significant.

**Table 7:** Odds ratios (ORs) and 95% confidence intervals (CIs) for the association of yearly nutrients intake from solanaceous vegetables and colorectal cancer risk.

**Yearly nutrients intake quartiles**	**Case**	**Control**	**Crude OR (95%CI)**	**Adjusted OR^a^ (95%CI)**
**Dietary fiber**				
Q1	274	208	1.00	1.00
Q2	234	209	0.87 (0.67,1.13)	0.80 (0.59,1.07)
Q3	167	208	0.57 (0.43,0.76)	0.58 (0.42,0.80)
Q4	158	208	0.52 (0.38,0.70)	0.50 (0.36,0.71)
P for Trend^b^			**<0.001**	**<0.001**
**Vitamin A**				
Q1	278	208	1.00	1.00
Q2	215	209	0.77 (0.59,1.00)	0.72 (0.53,0.96)
Q3	186	208	0.65 (0.50,0.86)	0.61 (0.45,0.83)
Q4	154	208	0.50 (0.37,0.67)	0.51 (0.36,0.71)
P for Trend			**<0.001**	**0.001**
**Vitamin E**				
Q1	283	212	1.00	1.00
Q2	213	212	0.76 (0.59,0.98)	0.66 (0.50,0.89)
Q3	178	203	0.63 (0.48,0.83)	0.58 (0.43,0.79)
Q4	159	206	0.52 (0.39,0.71)	0.51 (0.36,0.71)
P for Trend			**<0.001**	**<0.001**
**Vitamin C**				
Q1	263	209	1.00	1.00
Q2	196	208	0.74 (0.57,0.97)	0.68 (0.51,0.93)
Q3	207	212	0.76 (0.58,0.99)	0.68 (0.50,0.92)
Q4	167	204	0.60 (0.44,0.81)	0.51 (0.36,0.72)
P for Trend			**0.009**	**0.002**
**Folate**				
Q1	249	208	1.00	1.00
Q2	226	209	0.91 (0.70,1.19)	0.94 (0.70,1.26)
Q3	194	208	0.76 (0.58,1.00)	0.74 (0.55,1.01)
Q4	164	208	0.63 (0.47,0.84)	0.67 (0.48,0.93)
P for Trend			**0.011**	0.057
**Carotene**				
Q1	277	208	1.00	1.00
Q2	215	209	0.77 (0.59,1.00)	0.72 (0.53,0.97)
Q3	187	208	0.66 (0.50,0.86)	0.63 (0.46,0.85)
Q4	154	208	0.50 (0.37,0.68)	0.51 (0.36,0.72)
P for Trend			**<0.001**	**0.001**
**Vitamin B2**				
Q1	246	209	1.00	1.00
Q2	256	215	1.02 (0.79,1.32)	0.95 (0.71,1.27)
Q3	177	201	0.72 (0.54,0.95)	0.71 (0.51,0.97)
Q4	154	208	0.57 (0.42,0.77)	0.55 (0.39,0.77)
P for Trend			**0.001**	**0.002**
**Calcium**				
Q1	254	208	1.00	1.00
Q2	253	215	0.97 (0.75,1.24)	0.93 (0.70,1.23)
Q3	174	203	0.67 (0.50,0.88)	0.66 (0.48,0.91)
Q4	152	207	0.53 (0.39,0.72)	0.50 (0.35,0.71)
P for Trend			**<0.001**	**<0.001**
**Iodine**				
Q1	262	208	1.00	1.00
Q2	221	209	0.85 (0.65,1.11)	0.87 (0.65,1.18)
Q3	194	208	0.71 (0.54,0.94)	0.76 (0.56,1.04)
Q4	156	208	0.56 (0.42,0.75)	0.55 (0.39,0.76)
P for Trend			**0.001**	**0.004**

a *Adjusted by BMI, colon cancer in first-degree relative, smoking status, and alcohol drinking as category variables; and total energy and red meat intake as continuous variables*.

b *Test for trends calculated with the median intake of each category of nutrients intake as a continuous variable*.

*Bold values indicates statistical significance, which is defined as a two-side P < 0.05*.

## Discussion

Eggplant, potato, sweet pepper, and tomato are prevalent vegetables, especially in Northeast China. Therefore, we selected 833 matched case-control pairs from three hospitals in Northeast China for this case-control study. We did not find any correlation between the intake of total solanaceous vegetables and the risk of CRC, but we found that some certain solanaceous vegetable types, namely, eggplant, sweet pepper, and tomato had significantly inverse association with the risk of CRC. We also draw some conclusions that everyone knows that good dietary habits of having breakfast regularly, reducing fried food, barbecue, and hot and spicy foods; and increasing the intake of eggs, dairy products, fruits, and vegetables are beneficial to reduce the CRC risk.

In addition to some common chemical components in solanaceous vegetables, for example, anthocyanins and glycosidic alkaloids with an anti-cancer effect, each kind of vegetable itself also contains a unique substance that can play an anti-cancer effect. First of all, we guess that eggplant can reduce the incidence of CRC, which may be related to the following factors. Eggplant has been cultivated for hundreds of years, and China is its largest producer (29.5 million tons). Therefore, eggplant is a particularly important source of nutrients in the northeast part of China (29, 30). Studies showed that the extractive of eggplant fruit could protect DNA from injury, so eggplant may provide health benefits related to prevention or reduction of the risk of chronic diseases (e.g., cancer) (31). The fiber content in eggplant promotes digestion healthily and helps excrete waste and harmful toxins from the body to reduce the risk of colon cancer (32). Eggplant contains carotenoids. As antioxidants, carotenoids have the potential to reduce the risk of certain cancers, but high heat can reduce carotenoid levels (33, 34). This study also has confirmed that, except for the distal colon cancer subgroup, eggplants all show a prominent correlation with reduced CRC risk. The clinical and molecular characteristics of CRC vary with its anatomical location (35). Some dietary ingredients are more associated with distal colon and rectal cancer risk than proximal colon cancer (36). Currently, there are few research studies on the relationship between solanaceous vegetable intake and CRC; thus, the exact causes of the differences remain unclear.

Tomato might play a role in reducing the risk of CRC, and we guess the reason may be as follows. Studies have demonstrated that the lycopene in tomatoes can stop cells in the G0/G1 phase to inhibit tumor cell proliferation and induce apoptosis, which is also the basic reason for the anti-cancer effect of tomato (37). The overall single factor analysis and gender stratified analysis reveal that tomato intake is negatively correlated with cancer risk, but that it has no significant correlation with CRC risk after adjustment, which is consistent with an American study report suggesting that there is a statistically significant negative correlation between tomato consumption and colon cancer risk (38). A recent meta-analysis has assessed the relationship between lycopene intake and the risk of CRC. The relative risk of the highest category and the lowest category showed that lycopene intake had no significant correlation with the risk of CRC (RR = 0.94, 95% CI: 0.8–1.1). However, lycopene intake was significantly negatively correlated with colon cancer (RR = 0.88, 95% CI: 0.81–0.96) but showed no correlation with rectal cancer. The results of this study are consistent with the above: tomato intake is significantly related to the risk of proximal colon cancer with an OR value of 0.28 (95% CI = 0.1–0.89, *P* = 0.009) (39).

Although there were no significant differences in gender and subsite stratification analyses after adjustment, tomato also showed significantly negative association with CRC risk in the univariate analyses. Since the sample capacity was used in the subgroup analysis, we should treat these results cautiously.

The pectin in sweet pepper increases interleukin-6 in tumors and can regulate inflammation and angiogenesis to exert antitumor effects (40). Sweet peppers also contain capsaicin that can change the expression of several genes involving cancer cell survival, growth retardation, angiogenesis, and metastasis. Numerous research groups have found that capsaicin can exert anti-cancer effects through targeting multiple signaling pathways, oncogenes, and cancer suppressor genes in various types of cancer models (41–43), but the results of a case-control study in Sichuan province indicated that the consumption of hot peppers could neither increase nor decrease the risk of CRC (44). Many studies were targeted on red peppers or all pepper varieties currently. This study explicitly used sweet pepper as a research object, since it is the primary kind of pepper in the diet of people in Northeast China, which may be one of the reasons for the inconsistent results.

Also, this study indicated that there was no apparent correlation between potato intake and the risk of CRC through single-factor analysis, but the results showed that potato intake was positively correlated with the risk of CRC after adjustment. Despite a positive correlation among women and proximal colon cancer in the multivariate analyses, the gender stratification and subsite analyses found no significant results. A cohort study on Norwegian women also showed that large consumption of potatoes is associated with an increased risk of CRC (18). It was found in a case-control study that consumption of potatoes had a protective effect on rectal cancer in women but no association in men. In addition, the consumption of potatoes was also not associated with the risk of colon cancer (45). However, studies have also indicated that potatoes can provide essential nutritional antioxidants, such as vitamins, minerals, carotene, and polyphenols; and these substances can reduce cancer incidence. Simultaneously, the level of nutrients in potato is affected by different cooking and processing methods. Cooking potatoes under high temperatures will also produce acrylamide (a potential carcinogen) (46). The preparation of potatoes differs in thousands of ways worldwide, which is a relevant factor that needs to be considered when studying potato consumption and disease risk. There is no significant difference in the potato intake between the case group and the control group in this study; and cooking methods and other influencing factors are not discussed, which may explain the inconsistency of the research results.

We further investigated the common nutrients in solanaceous vegetables and found that some of their common chemical components can play a protective role. Solanaceous vegetables are rich in dietary fiber, and varieties of vitamins and minerals, so the protective effects of solanaceous vegetables on CRC may be attributed to the particular nutrients. Studies showed that a diet rich in fiber and folate, and high calcium and magnesium intake play a protective role in decreasing the incidence of CRC (6). A meta-analysis indicated that some food components, such as fiber, zinc, magnesium, vitamin A, vitamin C, vitamin E, folic acid, and carotene, may reduce the CRC risk because of their anti-inflammatory potential (47). Long-term observational studies suggested an inverse association between calcium intake and CRC risk (RR =.92 for 300 mg increase in total calcium every day) (48, 49). A recent case-control study has suggested a significant inverse association between dietary intake of folate and vitamin B2 and the risk of CRC, which is also in accordance with the results of this study (50). There is abundant evidence that these nutritional components from vegetables can impact on some cell functions, thereby decreasing the risk of CRC. Folate is important to reduce DNA uracil misincorporation so as to enhance genome stability. High levels of carotenoids and vitamins A, C, and E are able to reduce inflammation, genomic instability, proliferation, and increase apoptosis as well (51). In addition, high dietary fiber intake generates more butyrate, which may have anti-neoplastic properties, in colon by regulating immune response through suppressing histone deacetylation, also resulting in a protective effect against CRC (6).

The PDQ cancer information summary of NDI about CRC prevention states that the following factors increase the risk of CRC: age (>50), family history of CRC, personal history, inherited risk, alcohol (three or more alcoholic beverages per day), cigarette smoking, race (blacks), and obesity, while the following protective factors decrease the risk of CRC: physical activity, aspirin, combination hormone replacement therapy, and polyp removal (52). This study implied some additional suggestions on CRC prevention strategy. Specific solanaceous vegetables might be protective factors to CRC, particularly eggplant, sweet pepper, and tomato; therefore, increasing intakes of these individual solanaceous vegetables is more likely to lower the chance of developing CRC. However, regarding potato, we ought to decrease the consumption according to this study and current research studies discussed above.

As far as we know, this is the first epidemiological study on the intake of solanaceous vegetables and the risk of CRC in the Northeast of China. Since previous studies have mostly focused on the total vegetable intake or specific types of vegetables, this study classified these frequently consumed vegetables as Solanaceae vegetables for the first time. The particular advantages of this study are the very detailed food frequency data collected and a large number of cases. Therefore, we conducted deep analyses of the potential associations of specific solanaceous vegetable types with CRC risk. There have been several related studies (27, 53–56) that used this questionnaire in the past. The data of this study are extracted from the same questionnaire and have sufficient validity and reliability. We took the selected dietary intakes and dietary habits as potential mixed factors in the multivariate analyses, because different dietary habits may change the impact of solanaceous vegetables on CRC, resulting in a complex relationship between diet and CRC.

This research has some limitations. First of all, this study only collected dietary information of the chance of from the past 1 year. Compared with the intake in early life, this information may be less relevant to the risk of CRC. In response to this problem, we collect the information before any changes in dietary habits if there is, making the data more reflective of the relationship between long-term eating habits and the risk of CRC and making the results more convincing. Second, we have not considered the influence of the preparation method (raw, roasted or boiled, pickled) on food composition. Therefore, further research studies on food cooking methods and CRC risks should be carried out to predict the best way to prepare food. Besides, as recommended by the published literature, in consideration of the exploratory nature of this study, and we did not want to miss any potential meaningful findings, thereby, multiple comparison adjustments were not performed (57–59). In order to reduce recall bias, we chose newly diagnosed cases as research objects, because the remote diagnosis may result in reporting of newly changed behaviors as a consequence of the disease. Moreover, we tried to shorten the interval between the survey and the diagnosis as much as possible. The case group had to complete the questionnaire within 120 days from the day of diagnosis, and the control group accepted the interview within 3 months after the corresponding participants. In addition, specially trained personnel were arranged to carry out the structural questionnaire to the studied participants through a face-to-face interview. The participants were given enough time to recollect the eating pattern in their life history before answering (60). However, recall bias is inevitable in the form of a structured questionnaire. Selection bias cannot be ignored because of the hospital-based research design.

## Conclusion

In this research, no significant correlation was found between the intake of total solanaceous vegetables and the colorectal cancer risk in the northeastern Chinese population. Nevertheless, specific types of solanaceous vegetables were inversely associated with the CRC risk, indicating a protective effect, which could be further investigated through extensive cohort studies in the future.

## Data Availability Statement

The raw data supporting the conclusions of this article will be made available by the authors, without undue reservation.

## Ethics Statement

The studies involving human participants were reviewed and approved by Human Research Ethics Committees of The First Hospital, China Medical University, Shengjing Hospital of China Medical University, the First Affiliated Hospital of Dalian Medical University. The patients/participants provided their written informed consent to participate in this study.

## Author Contributions

ZL: conception, design, and providing critique. YL and SL: data analysis, interpretation, and preparation of draft manuscript. LJ: clinical data collection and compilation work. YZ: doing revisions. JS: clinical data collection, compilation work, and providing critique. All authors contributed to the article and approved the submitted version.

## Conflict of Interest

The authors declare that the research was conducted in the absence of any commercial or financial relationships that could be construed as a potential conflict of interest.
